# Micro-evolutionary divergence patterns of mandible shapes in wild house mouse (*Mus musculus*) populations

**DOI:** 10.1186/1471-2148-11-306

**Published:** 2011-10-18

**Authors:** Louis Boell, Diethard Tautz

**Affiliations:** 1 Max-Planck Institut für Evolutionsbiologie, August-Thienemannstrasse 2, 24306 Plön, Germany

## Abstract

**Background:**

Insights into the micro-evolutionary patterns of morphological traits require an assessment of the natural variation of the trait within and between populations and closely related species. The mouse mandible is a particularly suitable morphological trait for such an analysis, since it has long been used as a model to study the quantitative genetics of shape. In addition, many distinct populations, sub-species and closely related species are known for the house mouse. However, morphological comparisons among wild caught animals require an assessment in how far environmental and technical factors could interfere with the shape change measurements.

**Results:**

Using geometric morphometrics, we have surveyed mandible shapes in 15 natural populations of the genus *Mus*, with a focus on the subspecies *Mus musculus domesticus*. In parallel we have carefully assessed possibly confounding technical and biological factors. We find that there are distinct differences on average between populations, subspecies and species, but these differences are smaller than differences between individuals within populations. Populations from summer-dry regions, although more ancestral, are less distinct from each other than are populations from the more recently colonized northern areas. Populations with especially distinct shapes occur in an area of sympatry of *M. m. domesticus *and *M. spretus *and on recently colonized sub-antarctic islands. We have also studied a number of inbred strains to assess in how far their mandible shapes resemble those from the wild. We find that they fall indeed into the shape space of natural variation between individuals in populations.

**Conclusions:**

Although mandible shapes in natural populations can be influenced by environmental variables, these influences are insufficient to explain the average extent of shape differences between populations, such that evolutionary processes must be invoked to explain this level of diversity. We discuss that adaptive evolution may contribute to shape changes between populations, in particular in newly colonized areas. A comparison between inbred strains and wild mice suggests that the laboratory environment has no major systematic effect on the mandible shape and that such strains can be used as representatives of the natural shape differences between individuals.

## Background

Understanding the genetic basis of morphological phenotypic variation is a classical theme in evolutionary biology, with its roots going back to pre-Mendelian times. The problem is nowadays usually addressed within the framework of quantitative trait genetics and geometric morphometrics [1,2]. The mouse (*Mus musculus*) mandible has long been used as a model for approaching this question and associated evolutionary inferences [3-14]. While these quantitative genetic studies were conducted with laboratory strains, a desired long-term prospect in this field is to transfer the gained insights back to naturally evolved populations and to study the genetics of micro-evolutionary change in the wild.

There have been a number of comparative morphometric studies in wild house mice in different contexts [15-27], but except for [25], all of them were limited to specific instances of shape differences between small numbers of populations or species and most of them had a taxonomic focus. Not much is known therefore on how large the shape differences between populations within and between closely related species usually are, nor how they are distributed. However, such information is vital to ask the question which evolutionary forces act on shaping a morphological character, such as the mandible. While there is little doubt that purifying selection is required to maintain an optimal function of the mandible, very little is known in how far positive selection to new environmental conditions or neutral divergence processes influence mandible shape between populations and species. It is so far also unknown how variation among inbred strains relates to natural variation. Hence, to lay the foundations for studying micro-evolutionary divergence processes of the mandible and to eventually elucidate the genetic basis of shape differences, it is necessary to carefully record wild type variation in different populations and closely related species.

However, dealing with wild type variation of a shape component poses a specific challenge. It is to be expected that environmental influences, such as food, or biological variables, such as age and size, have an influence on shape as well. In addition, since it can be expected that the measured differences are subtle, technical error sources are also of concern, such as artefacts that could be generated by the preparation method or errors associated with the digitization process. Hence, it is necessary to address the question in how far all of these confounding factors could influence the measurements taken from wild caught animals.

While the relationship between mandible shape and ecological niche has been investigated for several taxa at the level of between-species diversity or higher [28-33], so far only one study conducted on shrews found intraspecific covariation between mandible shape and environmental variables [34]. In the mouse mandible, environmental influences have traditionally been investigated using hard diet vs. soft diet experiments, and diet consistency has indeed been found to influence mandible growth and shape [15,16,35-37]. However, the magnitude of the shape change introduced by the difference in diet consistency in relation to the shape differences between natural populations has not been addressed in these studies.

Here we have set out to assess the natural variation of mouse mandible shape using wild caught specimens (mostly from museum collections) and geometric morphometrics. The fundamental metric of shape difference in our study is Procrustes distance, which is the Euclidean distance between landmark configurations in the tangent shape space after Procrustes superimposition. This metric has the advantage that it is intuitive, as it is based on the tangent shape space (in contrast to Mahalanobis distances resulting from canonical variates analyses), and that it is comparable across experiments, if the shapes involved are not too different, which can be generally assumed for mouse mandibles.

As pointed out above, a crucial prerequisite for such a study is the assessment of technical error sources and we have therefore conducted a number of experiments specifically designed to get an estimate of the magnitude of the different confounding factors. We have then combined these in a statistical model to obtain an estimate of the likely combined influence of all these factors. We find that the effect of technical and biological error sources is on average smaller than the differences found between populations and species. This implies that evolutionary (genetic) divergence must be invoked to explain the level of phenotypic divergence among populations.

## Methods

### Samples

Our study covers 24 populations and strains of the subgenus *Mus *including wild-caught, captive and inbred mice (detailed in Table 1). 14 - 30 individuals were scored for each population or strain (Table 1). Most samples come from free-living mice, which were caught in the field. They were obtained either as loans from natural history museums (see additional file 1) or directly from colleagues. Museum samples were taxonomically re-analysed, based on the character list provided by Macholan [24]. Only unequivocally assignable material was used. The samples from Puente de Montañana (Spain) (from Senckenberg Museum, Frankfurt) turned out to be a mixture between *M. musculus *and *M. spretus*, which live sympatrically in this region. To assess interspecific divergence, we included two samples of each *Mus macedonicus *and *Mus spretus*. These species (together with *M. spicilegus*) are the closest sister species of *M. musculus *[38]. Only adult specimens were included in the study, juveniles being identified by the small size of their skull and mandibles and low tooth wear. Only right hemimandibles were considered, except in cases where only the left hemimandible of a specimen was undamaged. Museum specimens are usually prepared using either detergent solution or dermestid beetles, but the preparation method is mostly not documented. Hence, we assessed the influence of the preparation method on the shape measurements (see below).

**Table 1:** Population and strains used in this study

Name	Species	Geographical origin	Source	N
DOM EGYPT	*M. m. domesticus*	An Nawamis, Middle Egypt	ZFMK	29

DOM IRAN AHVAZ	*M. m. domesticus*	Ahvaz, South Iran	R.S.	19

DOM IRAN TEHERAN	*M. m. domesticus*	Teheran region, Iran	SMF	16

DOM SICILY	*M. m. domesticus*	Sicily	SMF	29

DOM SPAIN PUDEMONT	*M. m. domesticus*	Puente de montanana, Spain	SMF	26

DOM KERG GOUILLOU	*M. m. domesticus*	Kerguelen islands Gouillou	J.L.C.	27

DOM KERG COCHONS	*M. m. domesticus*	Kerguelen islands Cochons	J.L.C.	27

DOM GER MUNICH	*M. m. domesticus*	Munich, Germany	ZSM	14

DOM GER FRANKFURT	*M. m. domesticus*	Frankfurt, Germany	SMF	23

MUS HUNGARY	*M. m. musculus*	Hungary	SMF	25

CAS JOHNSTON ATOLL	*M. m. castaneus*	Johnston Atoll	NMNH	14

MAC GREECE	*M. macedonicus*	Chios, Greece	SMF	16

MAC TURKEY	*M. macedonicus*	Southwest Turkey	SMF	17

SPR SPAIN PUDEMONT	*M. spretus*	Puente de montanana, Spain	SMF	29

SPR SPAIN MADRID	*M. spretus*	Madrid, Spain	R.R.	28

LAB INBRED BALB/CBYJ	mixed *M. musculus *ancestry	undefined origin	JAX	30

LAB INBRED FVB/NJ	mixed *M. musculus *ancestry	undefined origin	JAX	30

LAB INBRED C57BL/10J	mixed *M. musculus *ancestry	undefined origin	JAX	30

LAB INBRED C57BL/6J	mixed *M. musculus *ancestry	undefined origin	MPIG	18

DOM WD INBRED STLT	*"M. m. domesticus"*	Straas, Germany	J.P.	20

DOM WD INBRED STRA	*"M. m. domesticus"*	Straas, Germany	J.P.	20

DOM WD INBRED STRB	*"M. m. domesticus"*	Straas, Germany	J.P.	20

CAS WD INBRED EIJ	*"M. m. castaneus"*	Thonburi, Thailand	MPIG	15

MUS WD INBRED PWD	*"M. m. musculus"*	Kunratice, Czech Republic	MPIG	20

LAB INBRED = laboratory inbred strains, WD INBRED = wild derived inbred strains, ZFMK = Zoologisches Forschungsmuseum Alexander Koenig, Bonn; SMF = Senckenberg Museum Frankfurt; ZSM = Zoologische Staatssammlung München; NHMN = National Museum of Natural History, Smithsonian Instititution, Washington (see additional file 1 for further details on museum samples); JAX = Jackson Laboratory, Bar Harbor; MPIG = Max-Planck Institute for Genetics, Berlin; R.S. = Rick Scavetta, J-L C. = Jean-Louis Chapuis, R.R. = Ruth Rottscheidt, J.P. = Jaroslav Pialek.

Five wild-derived inbred strains were studied. Three of them (Stlt, StrA, StrB [39]) are derived from *M. m. domesticus *and were obtained from the breeding facility in Studenec (Czech Republic). Their average ages at dissection were 21 weeks (ranging from 10 to 72 weeks), 23 weeks (ranging from 16 to 45 weeks) and 20 weeks (ranging from 9 to 33 weeks), respectively. Samples of PWD (*M. m. musculus*; JAX stock number 0046660; age at dissection 10 to 12 weeks) and Cast/EiJ (*M. m. castaneus*; JAX stock number 000928; age at dissection 9 to 12 weeks) were from the breeding facilities at the MPI for Genetics in Berlin.

Four sample sets represent classical inbred laboratory mouse strains. Three of them (BALB/cByJ, FVB/NJ, C57BL/10J) were obtained from the Jackson laboratory (Bar Harbor, USA) (age of dissection 10 weeks). An additional sample set of C57BL/6J mice (age at dissection 11 to 13 weeks) was obtained from the breeding facilities at the MPI for Genetics in Berlin.

To record a comparative developmental series, juvenile and adult specimens of C57Bl/6J and PWD were used. For C57BL6/J, 15 two weeks old specimens, 16 four weeks old specimens, and 17 six weeks old specimens were dissected and scanned. 16 eight weeks old specimens were scanned alive (anesthetized with Rompun/Ketamine). For PWD, 15 two weeks old specimens, 15 four weeks old specimens, and 16 six weeks old specimens were dissected and scanned. 18 eight weeks old specimens were scanned alive (anesthetized with Rompun/Ketamine).

To investigate age effects in adult mice, the following C57BL/6J mice were scanned alive (anesthetized with Rompun/Ketamine) at different ages, resulting in the following dataset: 16 eight weeks old animals, 10 ten weeks old animals, 10 twelve weeks old animals, 10 fourteen weeks old animals, 4 sixteen weeks old animals, 4 eighteen weeks old animals, 4 nineteen weeks old animals, 4 twenty weeks old animals, 4 twenty-one weeks old animals, and 5 twenty-three weeks old animals.

### Preparation methods

For the preparation with detergent solution, mouse heads were boiled for 1 hr in tap water, cooled for 1 hr and then incubated in a solution of 12 g/L commercial household detergent in tap water at 37°C for 3 days. The hemimandibles were then manually prepared from the heads and allowed to dry. For the preparation with dermestid beetles, mouse heads were pre-prepared by removing the fur and the brain, and the rest of soft tissue was removed by the beetles. The skulls were then frozen to kill remaining beetles and larvae, then thawed and allowed to dry. To investigate the effects of preparation on shape, skulls of 15C57BL/10J were prepared following each protocol. In addition, 20 animals of DOM IRAN AHVAZ were scanned alive (anesthetized with Rompun/Ketamine) and afterwards prepared using the detergent protocol.

### Diet effects

Two approaches were used to study diet effects. In the first we used laboratory outbred *M. m. domesticus *derived from animals trapped in the Massif Central in France [40] and fed them with soft diet versus hard diet. The hard diet animals (17 offspring from four breeding pairs) were fed on normal rodent pellets (Altromin standard diet No.1324). The soft diet animals (14 offspring from two breeding pairs) were fed on a powder diet, which has the same nutritional composition as the pellets (Altromin standard diet No. 1321). To ensure health of the mothers and to reduce maternal effects of the treatment, the food in the soft diet cages was changed from pellets to powder only 2 weeks after birth of the experimental animals, approximately two weeks before weaning. All mice in the hard diet/soft diet experiment were scanned alive (anesthetized with Rompun/Ketamine) at 8-10 weeks of age. Our second comparison was between wild caught animals (representing natural food conditions) and their first generation offspring raised under laboratory food conditions with pellets. For this we used 19 wild-caught *M. m. domesticus *from Ahvaz, Iran (DOM IRAN AHVAZ), and 24 F1 offspring of 6 breeding pairs of these mice. To quantify the impact of diet on Procrustes distances, we compared for both experiments the Procrustes distances of each "treatment" and "control" group to all of the wild mouse populations. Furthermore, we measured the Procrustes distances between "treatment" and "control" groups to assess the magnitude of distances caused solely by diet. Siblings were treated as statistically independent of each other, i.e. potential maternal or litter effects on shape would have been missed, but this makes the analysis more conservative.

### Data acquisition

**Figure 1:**
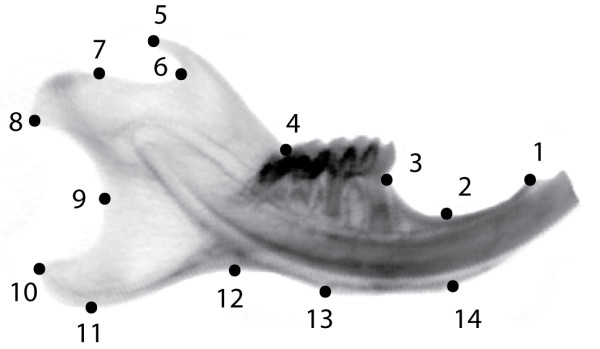
**Positions of the 14 landmarks used in this study on the outline of a mouse hemimandible radiograph**. LM1: Anterior terminus of bone dorsal of the incisor; LM2: Minimum of depression on dorsal side of incisor ramus; LM3: Bone/teeth transition anterior of M1; LM4: Intersection of ascending ramus with tooth row; LM5: Tip of processus coronoideus; LM6: Minimum of depression posterior to processus coronoideus; LM7: Anterior margin of condylar articular surface; LM8: Posteroventral tip of condyle; LM9: Minimum of depression formed by condyle and processus angularis; LM10: Posterodorsal tip of processus angularis; LM11: Posteroventral tip of processus angularis; LM12: Minimum of depression formed by processus angularis and incisor ramus; LM13: Posterior margin of muscle insertion area on ventral side of incisor ramus; LM14: Anterior margin of muscle insertion area on ventral site of incisor ramus.

All mandibles were scanned with a micro-computertomograph (microCT - VivaCT 40, Scanco, Bruettisellen, Switzerland). Whenever possible, the material was scanned "fresh", (either alive but anesthetized, or in a fresh, frozen/thawed cadaver, or in an ethanol-preserved specimen). The resolution of the scans depended on the material: 21 or 33 μm for prepared bone, 33 μm for alcohol preserved/fresh specimens, and 38 μm for anesthetized mice to keep the X-ray dosage low. In order to produce two-dimensional lateral views of the hemimandibles for geometric morphometrics, the scans were oriented as follows: hemimandibles were outlined in the tomographic slices, and the bone was segmented using a visually determined threshold of 230 mg HA/ccm (this unit is a standard measure of optical x-ray density of materials). From the triangulated surface of the three-dimensionally reconstructed hemimandible, the major spatial axis of the hemimandible were automatically determined as described in [41], and the 3D image datasets (both the original gray scale dataset as well as the segmented binary dataset) were then aligned in 3D with the direction of the major axis. From the aligned digital gray-scale dataset, a virtual 2D X-ray image was produced by adding the linear attenuation coefficients (the gray scale image values) in lateral direction (A. Laib, Scanco, Bruetisellen, pers. comm.). All these operations were done with the built-in software of the microCT system. Fourteen landmarks were digitized on each hemimandible using tpsDig2 [42] and tpsUtil [43], producing a set of 28 raw coordinates for each specimen. The landmarks assessed in this study are depicted in Figure 1.

### Statistical analyses

Most geometric morphometric analyses (except between-group PCA, see below) were performed in MorphoJ [44]. Analyses were carried out on different subsets of the material as described in results. The raw coordinates of each subset were subjected to a Procrustes fit in MorphoJ, whereby variation due to position, orientation and size was removed from the data, leaving only shape variation for further analysis. CVA (canonical variates analysis) was used to calculate the Procrustes distances between all samples in a given data subset (Procrustes distances are a part of the CVA output in MorphoJ). Discriminant function analysis was used as an additional tool for estimation of distinctness by numbers of misassigned specimens. Shape changes were visualized using the "warped outline drawing" function in MorphoJ. T-tests with sequential Bonferroni correction on Procrustes distances, calculation of Euclidean distances between shape change vectors and individual Procrustes coordinate configurations, and between-group PCA on Procrustes coordinates (using the bga function [45]) were performed in R [46]. Between-group PCA performs a PCA on group means and projects the data points of the individuals on the resulting axes. Procrustes coordinates were calculated in MorphoJ and between-group PCA using the bga function was then performed on Procrustes coordinates. Data plots were also produced in R. Summary statistics and Pearson correlation between Procrustes variance and fraction of specimens misassigned in DFA were calculated in PAST [47].

### Technical error measurements

To assess the magnitude of digitization error, we compared corresponding interpopulation Procrustes distances between two independent rounds of digitization. In order to reduce the impact of digitization error on the overall results, the average of both digitisations was used in the subsequent analyses. To assess the influence of sample size, we used samples with large numbers of specimens (more than 20), reduced them randomly to N = 15 and compared Procrustes distances before and after sample size reduction. To assess the influence of the preparation method, we scanned the same specimens before vs. after preparation and compared the respective sets of Procrustes distances to all of the wild mouse populations. We assessed also the influence of orientation of specimens inside the scanner by scanning a subset twice in random orientations.

### Simulation of the combined effects of technical errors and environmental factors

In order to assess how the technical error sources (digitization error, preparation method, small sample size) and the biological, non-genetic factors such as age, size and plasticity might add up to influence the final shape measurements, we ran the following simulation using R and MorphoJ. First, after a common Procrustes superimposition, we calculated the differences between the Procrustes mean shapes of the following seven sample pairs: DOM EGYPT digitization round 1 vs. DOM EGYPT digitization round 2 (digitization error), C57BL/6J unprepared vs. C57BL/6J prepared (preparation), DOM EGYPT all 30 specimens vs. DOM EGYPT reduced to 15 specimens (sample size), C57BL/6J 8-14 weeks old vs. C57BL/6J 16-23 weeks old (age), C57BL/6J with centroid size 524.77 - 565.68 vs. C57BL/6J with centroid size 565.72 - 587.63 (size), DOM IRAN AHVAZ from the field vs. F1 lab offspring of DOM IRAN AHVAZ (diet 1), *M. m. domesticus *hard diet vs. *M. m. domesticus *soft diet (diet 2). The resulting seven "error shape change vectors" were used to calculate 128 "total error vectors". Each "total error vector" was calculated in the following way: we assumed that each type of error could act in either direction, i.e. we multiplied each error shape change vector either by 1 or by -1. All 128 (= 2^7^) possible sign combinations were used. We further assumed that each error type would affect a given sample with varying strength. Therefore, we multiplied each error shape change vector by a coefficient randomly drawn from a normal distribution with mean = 0.5 and S.D. = 0.15. The mean value of this distribution was set to 0.5 because we the error shape change vectors have been constructed such as to correspond to the maximum impact of age, size, diet, preparation and sampling, respectively, and we considered it unrealistic that each population should always be affected maximally by each factor. S.D. = 0.15 was chosen to optimally cover the range between 0 and 1, whilst avoiding negative values. After multiplying each error shape change vector with a sign and a strength coefficient, all seven vectors were added together to give the total error vector. These vectors were used to assess the degree of between-population variation, which is likely to occur as a consequence of technical errors and non-genetic biological factors alone. To achieve this, we calculated the Euclidean distances between all total error vectors. The distribution of these error distances was then compared to the intra- and interspecific Procrustes distances between the populations of the wild mouse dataset using sequential Bonferroni corrected t-tests.

### Animal experimentation

All animal work followed the legal requirements, was registered under number V312-72241.123-34 (97-8/07) and approved by the ethics commission of the Ministerium für Landwirtschaft, Umwelt und ländliche Räume, Kiel (Germany) on 27. 12. 2007.

## Results

### Technical error sources

Our study combines samples from heterogeneous sources. It includes prepared skulls from wild-caught mice as well as ethanol-preserved specimens and live-scanned anesthetized mice. Also, the specimens were not always positioned in the same way inside the scanner and sometimes it is not easy to locate landmarks precisely and unambiguously on radiographs. Hence, it was necessary to assess the errors introduced by these technical factors. Because our metric of interest is Procrustes distances between populations, we quantified the effect of error sources on this metric.

The digitization error (measured as the difference between corresponding interpopulation Procrustes distances across two rounds of digitization) was found to be 4% on average (S.D. 4%, max16%). The error resulting from small sample size was 12% (S.D. = 11%, max 60%). The error introduced by orientation differences of specimens inside the scanner was negligible, i.e. not larger than expected from the error of digitization alone. The errors introduced through differences in preparation protocols (unprepared versus detergent or dermestid beetles) were on average 16% (S.D. = 6%, max 23%).

### Age, size and diet effects

Apparent differences between population samples in our wild mouse dataset could also be influenced by biological variables, such as systematic differences in age, size and environmental factors between populations. In this context, size could be a proxy for unknown environmental effects such as temperature, diet or habitat/population structure.

**Figure 2:**
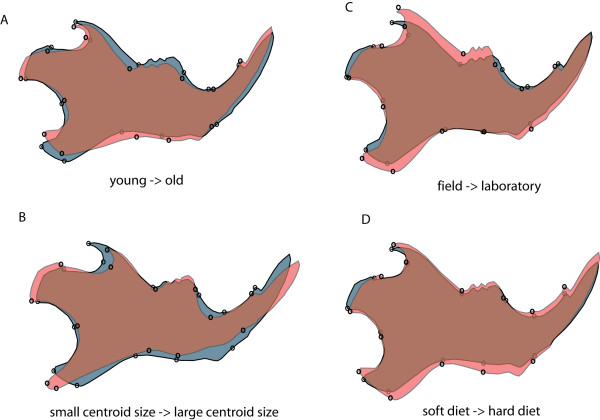
**Pair wise shape differences between four categories in grey and red shading, region of overlap in brown**. A) young versus old: Adult C57BL/6J mice 8-14 weeks old (gray shape) vs. 16-23 weeks old (red shape) (exaggerated x12); B) adult C57BL/6J mice with smaller (gray shape) vs. larger (red shape) mandibles (exaggerated x11); C) wild-caught Dom Iran Ahvaz (gray shape) vs. first generation offspring raised in captivity (red shape) (exaggerated x15); D) mice fed soft diet (gray shape) vs. hard diet (red shape) (exaggerated x3). Shape changes were calculated using discriminant function analysis between the respective groups.

To assess the impact of age, we divided our sample of 8-23 weeks old animals - an age span which corresponds roughly to the span expectation for adult wild mice [48] - into two age groups: 8-14 weeks old (N = 46) vs. 16-23 weeks old (N = 25). Our experiment thus mimics a strong systematic age difference between wild population samples. The average difference between corresponding Procrustes distances to wild mouse populations for young vs. old mice was 7% (S.D. 4%, max 15%) The Procrustes distance between the young and old group was 0.0205. The shape difference between these two groups is shown in Figure 2a.

To assess the impact of size differences on Procrustes distances, we used the same C57BL/6J mice as for the age experiment. This time, the mice were divided into two groups (each N = 31) according to centroid size (small mandibles: N = 31, mean = 556, S.D. = 9; large mandibles: N = 31, mean = 572, S.D. = 6). The average difference between corresponding Procrustes distances to wild mouse populations for large vs. small mandibles was 12% (S.D. 4%, max 18%). The Procrustes distance between the young and old group was 0.0137. The shape difference between these two groups is shown in Figure 2b.

For mice caught in the field versus their offspring raised on a laboratory diet, Procrustes distances to wild mouse populations differed on average by 18% (S.D. = 10%, max = 36%) with a Procrustes distance between treatments of 0.016. The shape difference between field and laboratory animals in this experiment are shown in Figure 2c. For the laboratory hard diet vs. soft diet comparison, they differed on average by 14% (S.D. = 8%, max = 30%) with a Procrustes distance between treatments of 0.0202. The shape difference between hard diet and soft diet animals in this experiment are shown in Figure 2d.

### Combined error and environmental factor estimate

**Figure 3:**
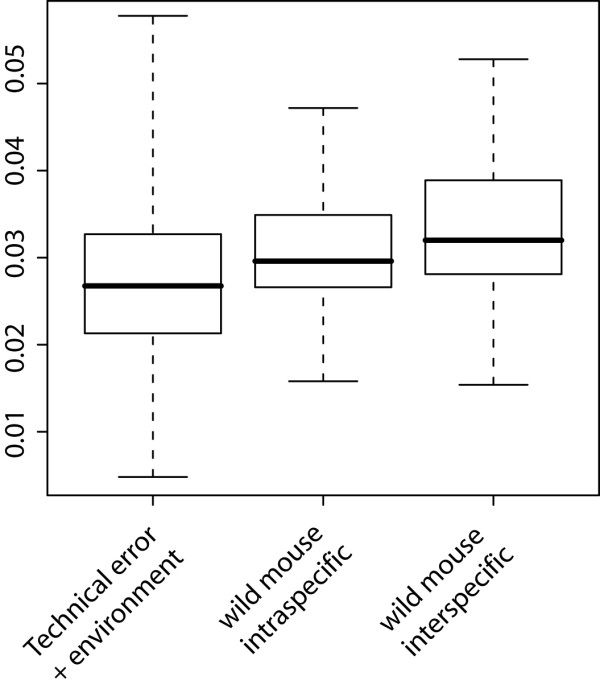
**Comparison of Euclidean distances between simulated technical error + environmental effect shape change vectors versus intra- and interspecific distances in our wild mouse reference dataset**. All three categories have significantly different means: simulation vs. intraspecific (p = 0.008); simulation vs. interspecific (p = 3.8 × 10^-8^); intraspecific vs. interspecific (p = 0.028); after sequential Bonferroni correction. Whiskers represent range of observed correlations.

To assess the combined effect of technical error and age, size and diet effects on the final measurements, we used the shape differences found in the experiments described above to simulate the distribution of Procrustes distances to be expected under various combinations of the respective factors (see Methods). We found that both, intraspecific and interspecific distances within the wild mouse dataset are on average significantly larger (p_intraspecific _= 0.008, p_interspecific _= 3.8^-08^, after sequential Bonferroni correction) than those created by the combination of all confounding factors (Figure 3). We conclude that technical error sources and non-genetic factors together are unlikely to fully explain the shape differences that we describe in the following in detail.

### Wild mouse diversity

**Figure 4:**
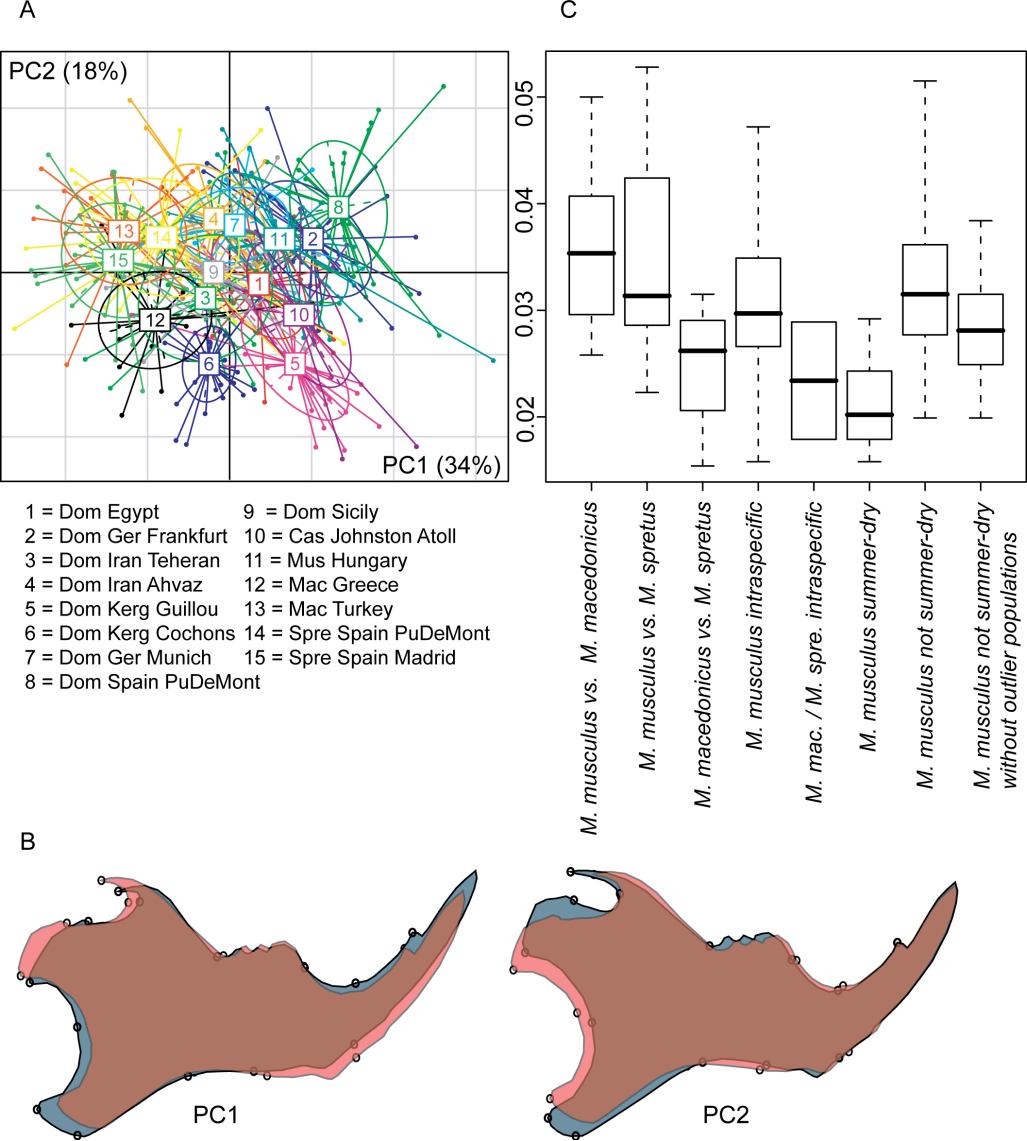
**Distances within the wild mouse reference dataset**. A) Between-group-PCA scatter plot of the first two PCs. B) Shape changes along PC1 and PC2 (from gray to red in positive direction). C) Distance categories - all pair wise distances within a category are plotted. Only the category "*M. mac */*M. spre *intraspecific" represents two comparisons - the lower point the *M. macedonicus *and the upper point the *M. spretus *intraspecific distances between the two populations each. Whiskers represent range of observed correlations.

In a first set of comparisons we focussed on the wild mouse diversity, i.e. mostly the samples from museums. We used two different representations to assess the differences in mandible shape in this wild mouse dataset, namely a between-group PCA plot for all specimens and comparisons between categories of distances (Figure 4). All Procrustes distances between population samples are listed in Table 2.

**Table 2:** Procrustes distances between the natural populations (× 10^-2^)

	(1)	(2)	(3)	(4)	(5)	(6)	(7)	(8)	(9)	(10)	(11)	(12)	(13	(14)
DOM EGYPT (1)														
DOM GER FRANKFURT (2)	2.9													
DOM IRAN TEHERAN (3)	1.8	3.5												
DOM SPAIN PUDEMONT (4)	3.9	2.9	4.7											
DOM IRAN AHVAZ (5)	2.4	3.4	2.9	3.2										
DOM GER MUNICH (6)	2.8	2.5	3.0	3.6	2.8									
DOM SICILY (7)	1.6	3.2	1.9	3.9	2.2	2.4								
DOM KER GUILLOU (8)	3.3	3.4	3.6	3.7	3.8	3.4	3.5							
DOM KER COCHONS (9)	2.9	3.9	2.7	4.3	3.2	3.9	2.9	2.7						
MUS HUNGARY (10)	2.3	2.0	2.8	3.1	2.7	2.4	2.5	3.5	3.5					
CAS JOHNSTON ATOLL (11)	2.5	2.8	2.9	3.8	3.5	3.8	3.0	3.2	3.0	2.5				
MAC GREECE (12)	3.0	4.2	2.6	5.1	3.6	3.5	2.7	3.4	2.9	3.8	3.6			
MAC TURKEY (13)	3.2	4.1	2.8	5.0	3.0	2.8	2.6	4.7	3.8	3.8	4.6	2.9		
SPRE SPAIN PUDEMONT (14)	3.1	3.6	2.5	4.3	2.7	3.0	2.6	4.1	3.1	2.8	3.8	3.1	2.6	
SPRE SPAIN MADRID (15)	3.1	4.3	2.2	5.3	2.9	3.1	2.4	4.5	3.4	3.6	4.3	2.7	1.5	1.9

The two first between-group PC axes explain 52% of variance between groups (Figure 4a). Axis 1 is mainly determined by the species differences between *M. musculus *(samples 1 - 11) and the two other species (*M. spretus *and *M. macedonicus; *samples 12 - 15). Three of the *M. m. domesticus *population samples are situated at the periphery of the range of natural variation along the major axes. One is a sample set that comes from a region of overlap with *M. spretus *(DOM SPAIN PUDEMONT - sample 8). This sample is distinct from other *M. musculus *along PC1, in the opposite direction to *M. spretus *and *M. macedonicus*. The shape change along this axis implies changes in the relative lengths of the angular process and the condyle (Figure 4b). The other two are from islands of the Kerguelen Archipelago (DOM KERG GUILLOU and DOM KERG COCHONS - samples 5 and 6) and represent very recent colonisations. These samples are distinct from other *M. musculus *along PC2, implying changes in the length of the coronoid process and the height of the posterior ramus (Figure 4b).

When comparing the inter- and intraspecific Procrustes distance categories (Figure 4c), it appears that the *M. musculus *populations are equally distant to *M. macedonicus *and *M. spretus*, while the latter two are closer to each other on average. Intraspecific distances within *M. musculus *appear to be much higher than within the two other species (note, however, that power is low for this comparison, because there are only two data points for intraspecific distances within *M. macedonicus *and *M. spretus*, hence the difference is not significant). When we focus on the *M. m. domesticus *populations from the summer-dry regions (DOM IRAN TEHERAN, DOM IRAN AHVAZ andDOM EGYPT), we find lower distances among them, when compared to the remainder of the *M. musculus *populations (p = 0.004 after sequential Bonferroni correction).

Stating that shape differences between population means exist, is not the same as stating that populations are distinct with respect to shape. To assess the degree of distinctness between populations within *M. musculus*, we performed a discriminant function analysis for each population against the pooled remaining wild-caught *M. musculus*. The results from cross-validation assessments of the number of misassigned specimens of the populations are shown in Table 3. While all populations were significantly distinct from the group formed of the pooled remaining wild-caught mice, on average 19% of the specimens were misassigned.

**Table 3:** Distinctness analysis of the natural populations

populations	% specimens misassigned	Procrustes variance (× 10^-3^)
DOM EGYPT	20.7	1.19
DOM GER FRANKFURT	17.4	1.37
DOM IRAN TEHERAN	31.3	1.77
DOM IRAN AHVAZ	21.1	1.4
DOM KERG GUILLOU	0	1.05
DOM GER MUNICH	21.4	0.92
DOM SPAIN PUDEMONT	7.7	1.4
DOM SICILY	24.1	1.44
DOM KERG COCHONS	3.7	0.75
CAS JOHNSTON ATOLL	28.6	1.92
MUS HUNGARY	28	1.84
		
**average**	**19**	**1.4**

### Wild versus inbred mice

Inbred mouse strains, which have previously been used for quantitative genetic studies of mandible shape, have evolved under laboratory conditions for numerous generations [34]. If this evolution or the inbreeding process had changed mandible shape, not only would this be of interest by itself, but it could also affect the evolutionary implications of such quantitative genetic studies. Therefore, we were interested to assess whether mandible shapes of inbred strains differ from the range of shapes found among wild mice.

**Figure 5:**
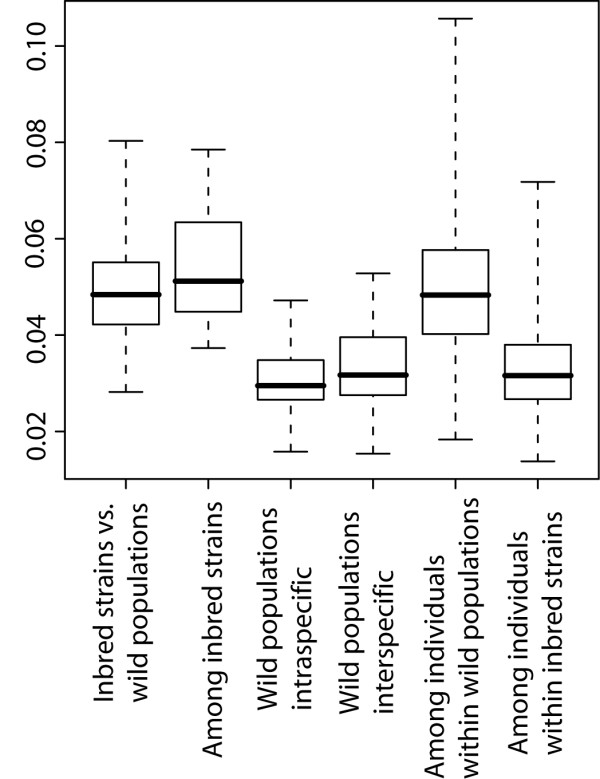
**Comparison of natural populations with inbred strains**. Procrustes distances between inbred strains and wild populations and among inbred strains compared to distances among wild populations (intra- and interspecific) and between individuals within populations. Whiskers represent range of observed correlations.

To compare mandible shape differences between inbred strains and wild populations, and among inbred strains, to natural variation between individuals within populations and between populations, we subjected inbred strains and wild mouse populations to a common Procrustes superimposition and then calculated the corresponding sets of Procrustes distances. The results are shown in Figure 5. It can be seen that the distances among inbred strains and between inbred strains and wild populations are much larger than the distances between wild populations, even across species boundaries (p = 2 × 10^-16^, 9 × 10^-12^, 2 × 10^-16^, and 3 × 10^-11^, respectively, after sequential Bonferroni correction). The phenotypic differences between inbred strains are therefore not comparable in extent to differences between natural populations. On the other hand, distances among inbred strains are not significantly different from distances between individuals within natural populations (p = 0.3 after sequential Bonferroni correction), which are much larger than the average differences between the populations themselves (p = 2 × 10^-16 ^after sequential Bonferroni correction). Therefore, the phenotypic differences between inbred strains correspond to the level of inter-individual rather than inter-population variation, with the latter being of minor importance as compared to the former.

**Figure 6:**
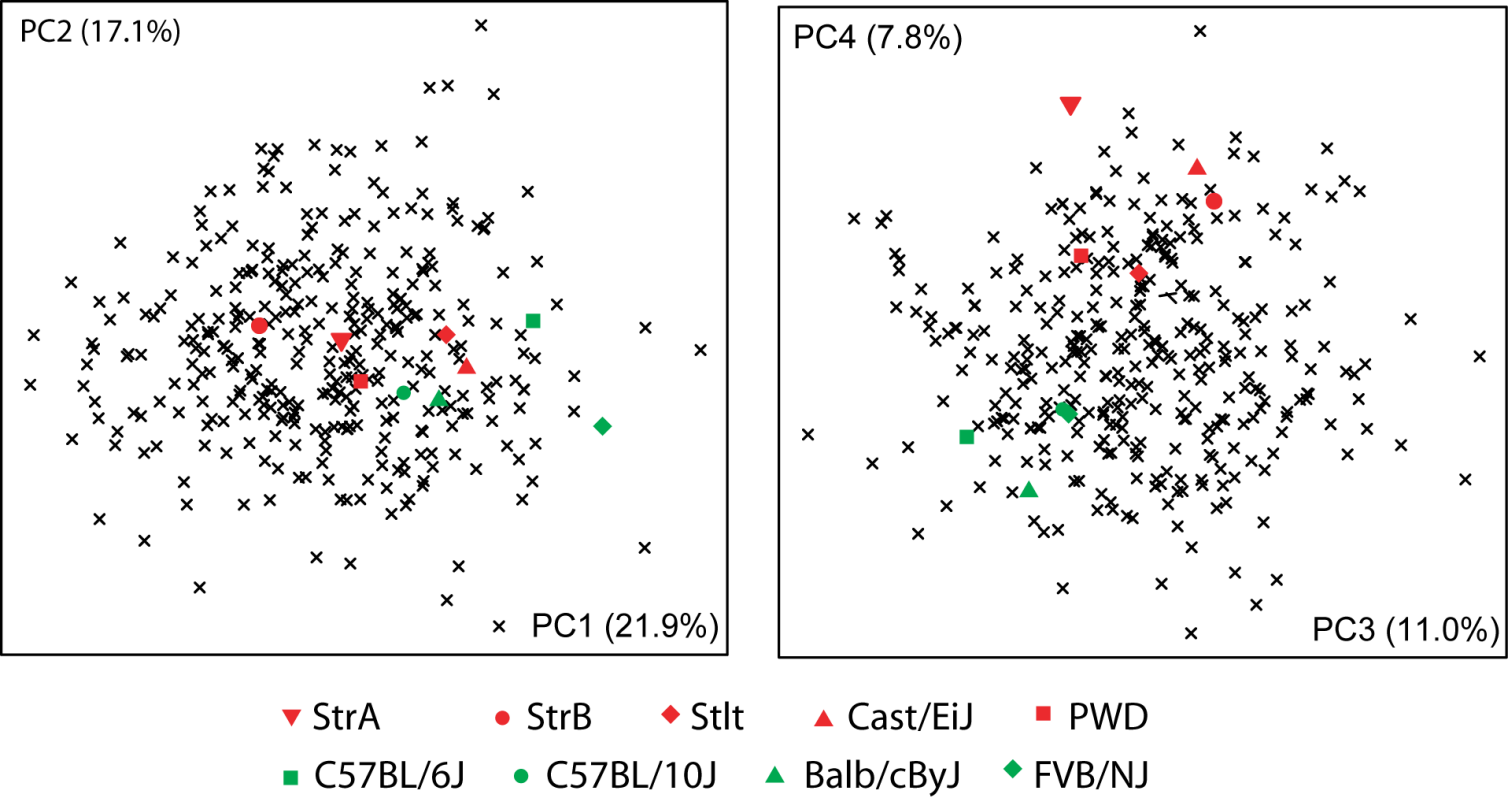
**Scatter plots of the first four axes of a PCA comparison of the aggregate shape space of all individuals from the natural populations with the Procrustes mean shapes of the inbred strains**. Red symbols: wild derived inbred strains; green symbols: classical inbred strains.

In order to assess the distribution of inbred strain shapes along the major axes of the space of natural inter-individual variation, we treated the inbred strains as if they were individuals (which is also justified by the fact that the are genetically homogeneous, i.e. represent only a single genotype) and calculated Procrustes mean shapes for them. These were then combined with the wild mouse dataset and a PCA was performed on this combined dataset. The results for the first four axes are shown in Figure 6. The inbred strain mean shapes are within the range of natural variation, although it seems that some patterns might occur (see discussion).

### Ontogenetic origin of shape differences between individuals

**Figure 7:**
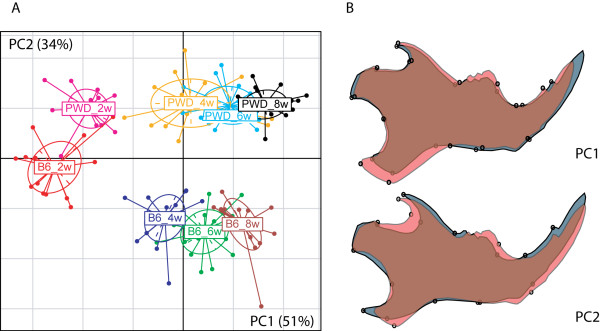
**Ontogenetic origin of shape differences between inbred strains**. A) scatter plot of the first two between-group PCs of two to eight week old mice of each C57BL/6J (B6) and PWD. B) shape changes along PC1 and PC2 (from gray to red in positive direction).

In the context of genetic differences in mandible shape, we were also interested to assess at which stage during ontogeny shape space differences would already become apparent. For this, we prepared a comparative ontogenetic series involving 2-8 weeks old mice from two inbred strains, LAB INBRED C57BL76J and MUS WD INBRED PWD. A between-groups-PCA of these samples (Figure 7) shows that the developmental trajectories of shape are running approximately parallel along PC1, and that some aspects of the characteristic shape difference between the lines are already present, albeit less pronounced, in the two week old animals of these strains. The processes, which lead to mandible shape differences between adults, thus appear to be initiated early in development, before the onset of major growth.

## Discussion

Knowledge of the natural variation of a trait is the basis for studying the microevolution of the trait. Our study aimed to assess the wild type shape space of the mouse mandible, which has become a major model for the evolution of morphological characters. However, dealing with wild-caught animals implies that these will differ with respect to age, size and previous diet. In addition, dealing with museum specimens could introduce further systematic differences caused by different preparation conditions, which are often not recorded. Apart of diet (see below) a systematic study on these possibly confounding factors has not been done before, but was evidently necessary, given that we record rather subtle differences. We have therefore first designed a number of tests to look at the influence of technical variables, such as preparation method of the skull, suboptimal sample size and digitization error. In addition we have looked at the influence of biological variables, such as age and size. The intuitively most important environmental factor that could influence mandible shape is diet and we have therefore paid particular attention to this. Previous studies on the influence of diet differences had found that food consistency has an effect on mandible shape [15,16,35-37]. But we have asked here for the first time how strongly these effects might confound measured differences between wild mouse populations. We found that there is indeed an effect of diet on mandible shape, but even in the extreme hard diet/soft diet shift experiment the effect is not more dramatic than for age or size differences, and the Procrustes distances between diet treatment groups are also smaller than the majority of intraspecific Procrustes distances. Furthermore, since house mice are generalists, which are not prone to specialize on any specific diet, we assume that extreme diet consistency differences are rare in nature (but see the case of Kerguelen mice below).

While each of these tests and experiments showed only a small influence on Procrustes distances between populations, it could well be that taking together all error sources and environmental factors would introduce more error in our data than any single error source. However, it is possible to use the shape differences between "treatment" and "control" groups in these experiments to calculate combined effects. We made use of this option to simulate a distribution of Procrustes distances under various combinations of error and environmental effects. This simulation is conservative in as far as it assumes cumulative effects of age, size, and diet treatments, whereas a smaller number of factors is likely to be relevant in real populations. Even under this conservative model, the combined error and environmental effect are unlikely to explain the average Procrustes distance between natural populations.

Given that the differences we have measured between the wild populations can apparently not fully be ascribed to technical and environmental factors, it seems safe to conclude that genetic factors contribute significantly to mandible shape differences. This conclusion is further supported by the fact that major shape differences exist between inbred strains that were derived from the same wild population at about the same time in the same laboratory (inbred strains Stlt, StrB and StrA, see below), i.e. the environmental influences should have been very similar in this case, but this is not the case: major differences are evident (see below). Furthermore, our assessment of the ontogenetic origin of shape differences between inbred strains suggests that they become manifest early in postnatal development, weeks before weaning, such that dietary differences acting later in life would only modulate already existing differences, rather than initiating them in the first place. Thus, the intraspecific diversity in mandible shape in the house mouse is apparently to a high degree genetically determined and can therefore be interpreted in an evolutionary context, involving an assessment of the role of selection and neutral evolution in shape divergence.

### The role of selection

Morphological traits in adults, such as mandible shape, are expected to be directly exposed to purifying selection, since their integrity should directly contribute to the fitness of the individual. This would imply that differences between populations should be driven by positive selection to new environmental conditions. However, neutral accumulation of differences over time would also be a possibility, in particular since there appears to be little gene flow even between populations of the same subspecies [40]. Some of the patterns that we see in our data allow to address this question.

The strongest indicator for selective constraints and only a small role for neutral divergence is the finding of relative higher similarity of shapes for *M. m. domesticus *populations from summer-dry habitats in Iran, Egypt and Sicily. This is of particular relevance, since these populations are at the same time the ones that are the relatively oldest ones. Based on molecular and fossil evidence, it has been shown that *M. m. domesticus *mice started to spread from the area of Iran into the Near East and Northern Africa approximately 8, 000 years ago, while the colonization of Sicily and Western Europe started only 3, 000 years ago [49]. Thus, *M. m. domesticus *has originated in a summer-dry climate and is expected to be optimally adapted to this. If one would assume a neutral divergence of shapes, one would expect that old populations are more different from each other than young populations, but this does not appear to be the case here. Instead, the younger populations in Western Europe show more morphological divergence than the older populations from Africa and the Near East. This divergence could be due to adaptive effects in the wake of colonizing new environments with new food sources, or could be due to the fixation of new alleles during the colonisation bottlenecks. At present it is difficult to distinguish between these possibilities, but one can point out that the molecular analysis of the populations has suggested that the colonisation bottleneck was not very strong [50].

The most divergent populations among *M. m. domesticus *are the ones that were caught on the sub-Antarctic islands of Guillou and Cochon, which are both part of the Kerguelen Archipelago where mice have arrived less than 200 years ago [51]. They occupy a new part of the shape space, indicated by their separation on the second PCA axis in comparison to the other wild-caught mice (Figure 4a). It is indeed known that these island mice have adapted to a new diet (preference for earth worms, which are otherwise not much used by mice [52], i.e. the common mandible shape difference along PC2 could have been caused by positive selection. On the other hand, this does not explain why they differ so much in PC1 (Figure 4a), although they live on ecologically very similar neighbouring islands. However, these mice came actually from two genetically distinct source populations [51], which could explain these differences. This would therefore be a case where parallel selection has led to some common changes (represented in PC2), but within different genetic backgrounds, which are responsible for the differences represented in PC1. While this interpretation is necessarily speculative (and does also not rule out additional bottleneck effects), it also corroborates the findings that we made in our companion paper on comparisons between mandible shape QTLs obtained from different experiments [53].

Another indicator of a possible influence of selection on shape comes from the finding that the *M. m. domesticus *population that lives in sympatry with a *M. spretus *population (DOM SPAIN PUDEMONT) shows a shift in shape space. In the PCA plot these mice are on opposite trajectories outside of the shape space of the other mainland *M. m. domesticus populations*, including the ones from the summer-dry regions, as well as from Germany (Figure 4a). The two species of mice included in this dataset were caught in the same small village in a single trapping campaign, suggesting that they share the same habitat. Hence, one could interpret the divergence of the *M. m. domesticus *shapes as a character displacement effect, in response to the interaction with *M. spretus*, which is the ancestral species in the region. However, this situation will need to be further analysed before a firm conclusion is possible, since it is so far not known in which form the two species might ecologically interact with each other.

### Inbred mice

To interpret shape differences between inbred strains in an evolutionary context, which means, with reference to natural variation, it is critical to understand which level of genetic diversity they reflect. Inbred strains are genetically comparable to individuals since they represent a single genotype only. Any variance between individuals from an inbred strain should therefore be ascribed to technical and environmental variance, while the strain mean shapes represent the genotype of these "individuals". We find that the within-strain variance of inbred strains is indeed much lower than the within-population variance of wild caught mice (Figure 5). Inbred strains represent thus essentially a random sample of chromosomes drawn from specific wild populations or admixed from various origins.

We find that inbred strains, which have been derived from wild populations, differ from each other more than the mean shapes between wild populations. This includes inbred strains, which have been derived from the same natural population, as is the case for the strains Stlt, StrA and StrB in our study. The Procrustes distances between these three strains (0.0373 - 0.0446) are as large as the larger interspecific distances between wild populations. The comparison of between- and within-population variation in wild mice (Figure 5) shows that inter-individual variation within natural population is much larger in comparison to differences between populations. The opposite pattern is seen for inbred mice (Figure 5) where the within strain differences are small due to inbreeding. The mandible shapes of inbred strains appear thus indeed to represent random samples of the original wild diversity, i.e. they "behave" as if they were individuals drawn from wild populations. This interpretation does of course not preclude possible additional changes due to inbreeding and laboratory evolution.

Although the mandible shapes of the inbred strains in our study are well within the range of natural variation, their distribution in Figure 6 suggests that there may be some pattern. The variation among inbred strains seems to be limited along PC2, while PC4 separates classical strains from wild-derived ones. While the former may be ascribed to laboratory effects, it is more difficult to speculate about a reason for this latter pattern. Perhaps it is partially due to phylogeny, since the classical strains have ultimately all been derived from the same admixed base population [54]. These questions will have to be revisited in the future.

## Conclusion

Variance comparisons within and between populations and closely related species are at the core of understanding micro-evolutionary divergence processes. We have shown here that this can also be applied to shape measurements in natural populations. Most importantly, we show that the unavoidable technical and biological variation in wild- caught samples is not as large as to hide underlying genetic patterns. Our analyses have identified some populations in which adaptive changes of mandible shape may have occurred. Two of these represent very recent invasions of islands, indicating that the adaptation can be fast, although mandible shape is controlled by many genes [9]. Our data provide also a solid basis for a further study of variance components and co-variance patterns (Boell, in preparation).

## Authors' contributions

LB and DT designed the study, LB did the measurements and the statistics, LB and DT wrote the manuscript. All authors have read and approved the final manuscript.

## Supplementary Material

Additional file 1 **Museum samples**. Collection numbers of museum material and museum addresses. Click here for file
