# Supplementary material for: Micro-evolutionary divergence patterns of mandible shapes in wild house mouse (Mus musculus) populations
Source: BMC Evol Biol. 2011 Oct 18;11:306. doi: 10.1186/1471-2148-11-306 (PMC3213108; doi:10.1186/1471-2148-11-306)
Supplement: Additional file 1 — Museum samples. Collection numbers of museum material and museum addresses. [file 1471-2148-11-306-S1.DOC]

Additional file 1

**Collection numbers of museum material and museum addresses**

Dom Egypt Dom Ger Frankfurt Dom Iran Teheran

(ZFMK) (Senckenberg) (Senckenberg)

99367 9321 46375

99436 9361 46376

99437 9389 46377

99439 9390 46378

99443 9392 46379

99446 9396 46381

99468 9466 46382

99472 9527 46383

99477 9793 46385

99480 9895 46386

99488 9932 46387

99497 9948 46388

99501 9970 46389

99502 9987 46390

99507 10018 46391

99509 10020 46392

99514 10021

99522 10023

99523 10029

99528 10263

99532 45123 Dom Spain PudeMont

99533 45124 (Senckenberg)

99534 45159

99537 31614

99543 31616

99548 31617

99549 31622

99553 31623

99558 31624

Mac Turkey 31625

(Senckenberg) 31626

Mac Greece 31630

(Senckenberg) 36663 31632

36664 31633

44931 36665 31634

44932 36666 31635

44935 36669 31636

44936 36670 31649

44937 36671 31650

44938 36672 31658

44939 36673 31659

44942 36674 31660

44943 36675 31661

44946 36677 31662

44947 36678 31664

44948 36679 31665

44951 36681 31666

44952 36682 31667

44953 36684 31749

Supplement 1 (continued)

Dom Sicily Spr Spain PudeMont Mus Hungary

(Senckenberg) (Senckenberg) (Senckenberg)

17332 31516 52150

17333 31612 52151

17335 31613 52152

17336 31615 52153

17337 31618 52154

17338 31619 52155

17339 31620 52156

17340 31621 52157

17341 31627 52158

17343 31629 52159

17344 31631 52160

17347 31637 52161

17348 31639 58228

17349 31640 58230

17350 31644 58231

17351 31645 58233

17352 31646 58234

17353 31647 58234

17354 31651 58235

17355 31652 58236

17356 31654 58238

17357 31656 58239

17358 31657 58241

17361 31744 58242

17362 31745 58243

17363 31746

17364 31747

37020 31748

37022

Cas Johnston Atoll Dom Ger Munich

(Smithsonian) (ZSM)

360999 All specimens 1977 Kleinlangenheim,

361001 no individual numbers available.

361002

361003

361004

361005

361008

361009

361010

362132

362135

362137

362141

362143

Supplement 1 (continued)

**Adresses of the museums**

Senckenberg:

Senckenberganlage 5

D-60325 Frankfurt am Main

Germany

ZSM:

Zoologische Staatssammlung München

Münchhausenstrasse 21

D-81247 München

Germany

ZFMK:

Zoologisches Forschungsmuseum Alexander König

Adenauerallee 160

D53113 Bonn

Germany

Smithsonian:

Smithsonian Institution

National Museum Of Natural History

Department of Vertebrate Zoology

Division of Mammals

NHB MRC 108; 10th & Constitution Ave,N.W.

Box 37012

Washington, DC 20013-7012

USA
